# A Five-Biomarker IHC-Based Signature Predicting Outcome in Breast Cancer Patients Following Adjuvant Anthracycline-Based Chemotherapy

**DOI:** 10.3390/cancers18071092

**Published:** 2026-03-27

**Authors:** Siyao Wang, Elaine Gilmore, Syed Umbreen, Cory Fines, Roberta Burden, Stephen McQuaid, Niamh Buckley

**Affiliations:** 1 School of Pharmacy, Queen’s University Belfast, Belfast BT9 7BL, UK; swang22@qub.ac.uk (S.W.); e.gilmore@qub.ac.uk (E.G.); s.umbreen@qub.ac.uk (S.U.); cory-fines@uiowa.edu (C.F.); r.burden@qub.ac.uk (R.B.); 2 Department of Radiation Oncology, University of Iowa, Iowa, IA 52242, USA; 3 Johnston Cancer Research Centre, Queen’s University Belfast, Belfast BT9 7AE, UK; cancers@mdpi.com

**Keywords:** breast cancer, biomarker signature, adjuvant chemotherapy

## Abstract

**Background/Objectives**: Breast cancer remains the leading cause of cancer-related death among women worldwide. While tools such as Adjuvant Online, PREDICT, OncotypeDx and Mammoprint identify patients at higher risk of relapse who should therefore be offered chemotherapy, there are currently no tools to accurately predict response to chemotherapy, with varied response rates (regardless of subtypes, etc.) of 8–70% reported. Accurately stratifying patients based on their likelihood of benefiting from SoC chemotherapy is therefore critical to guide personalised treatment decisions. **Methods**: A retrospective cohort of 293 breast cancer patients treated with SoC adjuvant anthracycline-based regimen was analysed. Five biomarkers (TOP2A, PTEN, EGFR, IGF1R, and phospho-mTOR), selected for their prognostic and therapeutic relevance, were assessed using immunohistochemistry (IHC) combined with digital pathology. **Results**: Biomarker expression was quantified using the digital pathology platform, QuPath, with each marker, when stratified based on high/low expression, demonstrating a significant association with relapse-free survival following SoC chemotherapy in specific subtypes of breast cancer. A composite five-biomarker signature was then generated by integrating the individual biomarker scores to improve prognostic precision. Patients with a five-biomarker signature score greater than zero exhibited a significantly higher likelihood of favourable outcomes following anthracycline-based chemotherapy compared with those with a score of zero or below. **Conclusions**: This study establishes a novel IHC-based five-biomarker signature capable of predicting patient outcome in the context of SoC chemotherapy. As the signature relies exclusively on IHC, it is simple, cost-effective and readily integratable into routine diagnostic workflows. In addition to its prognostic value, several biomarkers within the panel are potentially actionable, offering opportunities to guide targeted therapies in patients predicted to have poor response to conventional chemotherapy.

## Simple Summary

Breast cancer remains a leading cause of cancer-related death among women, with accurate prediction of treatment response being crucial for personalised treatment and improved outcomes. This study investigates the prognostic relevance of five biomarkers (TOP2A(Topoisomerase II alpha), PTEN (Phosphatase and TENsin Homologue), EGFR (Epidermal growth factor receptor), IGF1R (Insulin-like Growth Factor 1 Receptor), and phospho-mTOR (Mechanistic Target of Rapamycin)) in a retrospective cohort of 293 breast cancer patients treated with standard of care (SoC) adjuvant anthracycline-based chemotherapy. The expression of these biomarkers was quantified using digital pathology revealing significant associations with favourable relapse-free survival across specific and varied breast cancer subtypes. A composite five-biomarker score was developed, and patients with a high score had a significantly higher likelihood of relapse-free outcomes following chemotherapy compared to those with a lower score. This study presents a novel, protein-based signature that can predict chemotherapy response and suggests potential for guiding targeted therapies in patients who are less likely to respond to standard treatment.

## 1. Introduction

Breast cancer is a highly heterogeneous disease, characterised by its varied responses to standardised treatment regimens, underscoring the need for biomarkers to stratify patients and optimise treatment based on tumour biology. Current management relies on well-established biomarkers, including oestrogen receptor (ER*α*), progesterone receptor (PR), and human epidermal growth factor receptor 2 (HER2), which guide the use of endocrine and HER2-targeted therapies, respectively [1,2]. In addition to this, predictive tools such as Adjuvant Online, PREDICT, Oncotype DX and MammaPrint have been developed to estimate the risk of recurrence and guide decisions on the need for chemotherapy [3,4,5,6]. However, a considerable proportion of patients, who are deemed at high risk of relapse based on these tools and therefore receive chemotherapy, do not derive significant benefit from the treatment. For example, the pathological complete response (pCR) rate to anthracycline-based chemotherapy has been found to be approximately 15% following neoadjuvant chemotherapy in breast cancer as a whole [7]. In hormone receptor-positive/HER2-negative breast cancers, combined with anti-oestrogen therapy, pCR rates generally range from 8 to 20% with anthracycline-based chemotherapy [8,9]. In HER2-positive tumours, pCR rates of 18–47% have been reported when anthracycline–taxane regimens are combined with HER2-targeted therapies [10]. In Triple Negative Breast Cancer (TNBC), despite being associated with the highest mortality rates, pCR rates of approximately 35–70% have been observed, highlighting the “TNBC paradox” [11]. Crucially, reliable biomarkers to predict which patients will benefit from chemotherapy are lacking, leaving many patients exposed to substantial toxicities without a clear expectation of clinical benefit [12]. This gap highlights the urgent need for additional biomarkers to predict patient outcome with chemotherapy and enable more personalised therapeutic strategies.

Previous work, led by our collaborator, Professor Salto-Tellez, selected a panel of biomarkers that had differential expression between normal tissue and invasive lesions, prioritising those with prognostic potential and therapeutic relevance [13]. From this, five biomarkers, TOP2A, PTEN, EGFR, IGF1R, and mTOR (specifically the phosphorylated form Ser2448), were selected for further investigation.

The first collaborative study evaluated these biomarkers in pre-invasive lesions to determine whether their expression could distinguish lesions likely to progress to invasive cancer, with the study comparing biomarker expression between purely non-invasive lesions (PNLs) and those with co-existing invasive cancer (CEIN) as a surrogate indicator for progression, as well as across normal, benign, in situ, and invasive tissues [14]. High TOP2A expression was associated with high-grade invasive lesions, while PTEN loss was most frequent in CEIN, particularly in high- or intermediate-grade ductal carcinoma in situ (DCIS). EGFR expression level was higher in DCIS and invasive carcinoma than in benign lesions, with most EGFR-positive DCIS being high or intermediate grade. IGF1R loss occurred in 11% of DCIS cases, predominantly high-grade, and was associated with the negative prognostic feature, comedonecrosis. p-mTOR overexpression was frequent in pre-invasive lesions and also correlated with comedonecrosis. Collectively, these findings suggested that this five-biomarker panel could help refine patient stratification and management. Further evaluation of the same panel in invasive breast cancer may therefore enhance its clinical relevance and support more comprehensive therapeutic decision-making [14]. Consequently, in this study, we hypothesised that the same five biomarkers could be used to improve personalised treatment strategies for early breast cancer by investigating the prognostic relevance of the biomarkers in the context of anthracycline-based chemotherapy treatment.

## 2. Materials and Methods

### 2.1. Tissue Microarray (TMA) and Immunohistochemistry (IHC)

The Breast 300 cohort has been used in numerous studies and described previously [15,16,17,18,19,20]. Briefly, patients received total or partial mastectomies with axillary node clearance during the period between September 1997 and May 2009 and received adjuvant anthracycline-based chemotherapy, with or without radiotherapy after the surgery. Guided by hormone receptor and HER2 status, hormonal therapy and trastuzumab were applied, respectively, following standard of care. None of the patients underwent neoadjuvant treatment (Table 1). Tissue was obtained through the Northern Ireland Biobank with appropriate ethical approval (NIB ref.: 12-00017). From the total of 303 identified patients, slides and corresponding paraffin blocks were available for 293 patients. All the slides were reviewed through H&E staining for tumour block selection by an experienced breast histopathologist. A manual tissue arrayer (Beecher Instruments, Silver Spring, MD, USA) was used to construct the TMA, extracting tissue cores 1 mm in diameter from donor blocks to recipient blocks as described [21]. Each patient was represented in triplicate on the TMA.

All IHC was performed by the Northern Ireland Molecular Pathology Laboratory, which holds UK Clinical Pathology Accreditation. H&E staining was first performed within the sections to evaluate the TMA integrity and tumour representation. Sections measuring 4 μm in thickness were cut from the TMA blocks using a rotary microtome and dried overnight at 37 °C before undergoing IHC staining using an automated immunostainer (Leica Bond-Max, Milton Keynes, UK). The antigen-binding sites were detected using a polymer-based detection system (Bond, Newcastle Upon Tyne, UK, Cat. No. DS 9800). All biomarkers were evaluated on appropriate control tissues and reviewed by an experienced pathologist, Prof. Manuel Salto-Tellez, and an immunohistochemist, Dr. Stephen McQuaid. The clone, optimised antibody conditions and automated platform information are summarised in Table 2. Visualisation of the IHC staining was achieved with diaminobenzidine, counterstaining with haematoxylin and mounted in DPX for preservation and subsequent analysis [14]. The images of the tissues were scanned by the Aperio ScanScope CS (Leica Biosystems, Milton Keynes, UK) at ×40 magnification in the Northern Ireland Molecular Pathology Laboratory.

### 2.2. Gene Expression Profiling

The extraction of total RNA from formalin-fixed paraffin-embedded (FFPE) tumour samples from the same Breast 300 cohort was carried out using the Roche High Pure RNA Paraffin Kit (Roche Diagnostics, Burgess Hill, UK), as described previously [22]. The amplification of total RNA was conducted utilising the NuGEN WT-Ovation FFPE System (NuGEN, San Carlos, CA, USA), followed by hybridisation to the custom-designed microarray, Almac Breast Cancer DSA (Affymetrix, Santa Clara, CA, USA). A normalised gene expression matrix of 40,722 probesets and 275 patients was obtained using the R package “rma” [16].

### 2.3. Image Analysis

Access and analysis of images were carried out under appropriate ethical approval (MHLS20_43). Digitised TMA images were imported and analysed by QuPath [23]. Scanned TMAs were dearrayed to identify and outline each core, with manual QC ensuring no overlap. Cores with fewer than 100 tumour cells were excluded, and tissue detection removed areas of shadows, necrosis, and folds. Cells were detected using a user-optimised threshold, and tumour and stromal cells were classified via machine learning. Positive cells were identified based on DAB optical density thresholds. The percentage and intensity of positive cells were exported to Excel, and, following quality control with manual review if required, correlated with clinical data.

### 2.4. Breast Cancer Subtypes

Breast cancer was classified into five subtypes according to St Galen subtypes based on ER, PR, HER and Ki67 staining performed on the TMA [24]: Luminal A (ER+ and/or PR+, HER2−, Ki67−), Luminal B HER2-negative (ER+ and/or PR+, HER2−, Ki67+), Luminal B HER2-positive (ER+ and/or PR+, HER2+), HER2-enriched (ER−, PR−, HER2+), TNBC (ER−, PR−, HER2−). ER and PR expressions were considered as positive when the proportion score ≥ 2 and the total score ≥ 3 using Allred score [25]. This threshold equates to at least 1% of cells exhibiting nuclear expression of any intensity. Ki67 expression was considered as positive when the proportion score > 14% [26].

### 2.5. Scoring and Assessment

A Histochemical score (H-score) was used to quantitatively assess the expression of the protein in IHC. The staining intensity was scored between 0 and 3 or, where indicated, 0 and4. The final H-score was calculated by the following formulation: H-score = (1 × % of cells with 1 intensity) + (2 × % of cells with 2 intensity) + (3 × % of cells with 3 intensity) + (4 × % of cells with 4 intensity)

Statistical analysis was conducted using GraphPad Prism 9.5.1 (GraphPad Software, San Diego, CA, USA). The Mann–Whitney test was applied to compare two independent groups. The Kruskal–Wallis test was used for multiple-group unpaired comparisons, followed by post hoc Dunn’s correction, unless otherwise specified. Kaplan–Meier survival curves were conducted using the Log-rank (Mantel–Cox) test to assess survival differences (Log-rank *p* value) and estimate Hazard Ratios (Log-rank HR) in both GraphPad Prism and Cutoff Finder (https://molpathoheidelberg.shinyapps.io/CutoffFinder_v1/, accessed on 25 September 2023) [26]. Multivariate analysis was conducted by Cox proportional hazards regression.

## 3. Results

### 3.1. TOP2A, PTEN, IGF1R, EGFR and p-mTOR Protein Expression Significantly Varied Between the Subtypes in Breast Cancer

**Figure 1:**
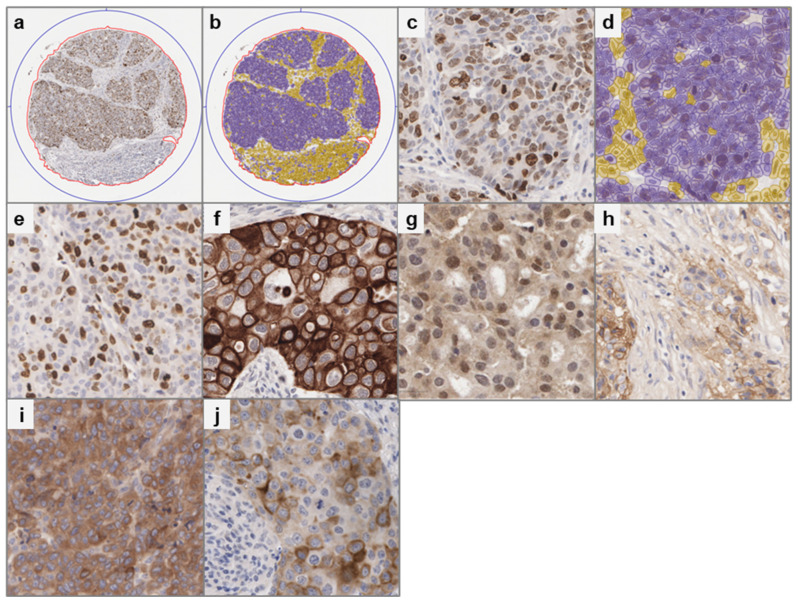
Representative IHC staining of five biomarkers in the Breast 300 cohort. Haematoxylin staining (blue) indicates the cell nucleus and DAB staining (brown) indicates biomarker expression. IHC staining with cell classification applied (**a**) in the whole core with the magnification × 5 (**b**) with the magnification × 40 and with QuPath mask (**c**) in the whole core with the magnification × 5 (**d**) with the magnification × 40. Purple = tumour cells, Yellow = stromal cells. Homogenous staining observed in (**e**) TOP2A and (**f**) peri-nuclear p-mTOR. Heterogeneous staining observed in (**g**) PTEN (nuclear), (**h**) EGFR, (**i**) IGF1R and (**j**) whole tumour cell p-mTOR.

To investigate the role of each of these biomarkers following anthracycline-based chemotherapy in the early-stage, high-risk breast cancer patients, a previously described breast cancer TMA [15,16,17,18,19,20] was stained for IHC-based assessment. In order to facilitate robust digital scoring, manual review of staining, in conjunction with known biomarker expression/function, was used to determine the most appropriate regions within a given core to score. As expression of each of the biomarkers was predominantly restricted to tumour cells, a tumour/stroma classifier was applied to allow quantification of expression within the tumour regions only (Figure 1). Next, if applicable, scoring was restricted to the specific localisations within the tumour cell in line with the QuPath workflow. For example, nuclear staining was selected to quantify TOP2A and PTEN, while whole-cell quantification was used for the expression of EGFR, IGF1R and p-mTOR. A second method was also applied to p-mTOR with perinuclear staining captured specifically to reflect its presence in the mTORC1 complex (Table 3). Next, a scoring method was determined based on staining patterns, using either the percentage of positive tumour cells or a H-score. TOP2A and perinuclear p-mTOR exhibited homogeneous staining intensity within each patient core; therefore, only the percentage of positive tumour cells was quantified. In contrast, PTEN, EGFR, IGF1R, and whole-cell p-mTOR showed heterogeneous staining intensity across tumour cells, so both the percentage and intensity of positivity were incorporated using a H-score (Figure 1).

**Figure 2:**
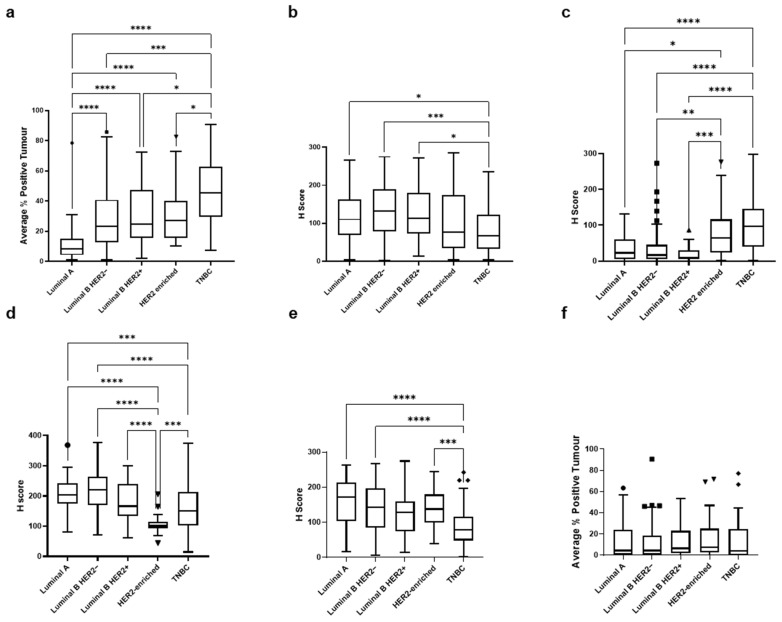
IHC score of the five biomarkers between breast cancer subtypes. Box and whisker plot showing protein expression of (**a**) TOP2A in tumour nucleus (*N* = 273), (**b**) PTEN in tumour nucleus (*N* = 265), (**c**) IGF1R in tumour cells (*N* = 268), (**d**) EGFR in tumour cells (*N* = 271), (**e**) p-mTOR in tumour cells (*N* = 281) and (**f**) p-mTOR in peri-nucleus (*N* = 281). Significance was determined using a Kruskal–Wallis analysis with post hoc Dunn’s correction for multiple testing. * *p* < 0.05, ** *p* < 0.01, *** *p* < 0.001, **** *p* < 0.0001.

These parameters were then applied to all patient samples for each biomarker with a range of expression/scores observed (Figure S1), which varied significantly between the molecular subgroups of breast cancer (TOP2A, EGFR, IGF1R and p-mTOR whole-cell staining: *p* < 0.0001; PTEN: *p* = 0.0006), except p-mTOR peri-nuclear expression which showed consistent expression across the subgroups (Figure 2). Both TOP2A and EGFR showed the significantly highest expression in the TNBC, while PTEN and whole-cell expression of p-mTOR showed the significantly lower expression in the TNBC.

### 3.2. TOP2A, PTEN, IGF1R, EGFR and p-mTOR Expression Correlate with Outcome Following Anthracycline-Based Chemotherapy

To investigate potential prognostic significance, expression of each of the biomarkers was correlated with outcome. Relapse-free survival was used as the primary endpoint, as it has been shown to be an accurate predictor of overall survival, given that there is no cure for secondary BC [27]. Given the absence of clinically defined thresholds for these biomarkers, a standardised analytical framework was applied across all five markers to evaluate multiple candidate cutoff strategies. This approach ensured that threshold selection followed a consistent and systematic process, while allowing identification of the most clinically meaningful stratification for each biomarker. If, when patients were classified as having high or low biomarker expression based on the median expression value within each molecular subtype, a significant association with outcome was observed, this was selected, given it represents an unbiased approach. In addition, potential thresholds were also investigated using the online tool Cutoff Finder [28], which identifies the threshold with the most statistically significant survival association. If the identified threshold was notably different to the median in terms of value and Hazard ratio/*p*-value, this was instead selected. Due to the heterogeneous expression patterns of these biomarkers across different molecular subtypes, patient outcome was also assessed separately within each breast cancer subtype (Tables S1–S5, Figures S2 and S3). Subtypes exhibiting a similar pattern and similar biological relevance were subsequently combined into a single group with the threshold representing the best biological significance selected if required (Tables S1–S5, Figures S2 and S3). High expression of each of the biomarkers was associated with improved outcome, but only in distinct patient subgroups; high TOP2A expression in the ER-negative subgroup, high PTEN expression in the HER2-positive and Luminal B HER2-positive subgroup, high IGF1R expression in the Luminal B HER2-positive subgroup, high p-mTOR (whole-cell expression) in the Luminal B subgroup and p-mTOR (peri-nuclear expression) in the HER2-positive group (Table 4). While no obvious correlation was observed between EGFR expression and outcome, it was noted that rare cases displayed extreme positive expression and therefore the clinical relevance was investigated. Results suggested that extremely high EGFR expression could be associated with better RFS in all breast cancer subtypes except Luminal A although this did not reach statistical significance. In addition, multivariate Cox regression analysis provided further evidence that all five biomarkers, within their corresponding breast cancer subtypes, may serve as independent prognostic markers (Table S6).

### 3.3. A Combined Five-Biomarker Signature Can Predict Patient Outcome in the Context of Anthracycline-Based Chemotherapy Treatment

A combined approach was then considered to provide broader clinical usefulness by allowing the biomarkers to stratify patients collectively rather than individually. In order to develop a combined signature, a simple approach was taken whereby if a patient was assigned high for a given individual biomarker a score of +1 was assigned while −1 was assigned for low expression given all biomarkers were associated with improved outcome with high expression. The final signature score was then calculated as the sum of these scores for the five biomarkers, resulting in a possible range from −6 to +6.

Signature Score = TOP2A + PTEN + EGFR + IGF1R + p-mTOR (whole cell) + p-mTOR (peri-nucleus)

**Figure 3:**
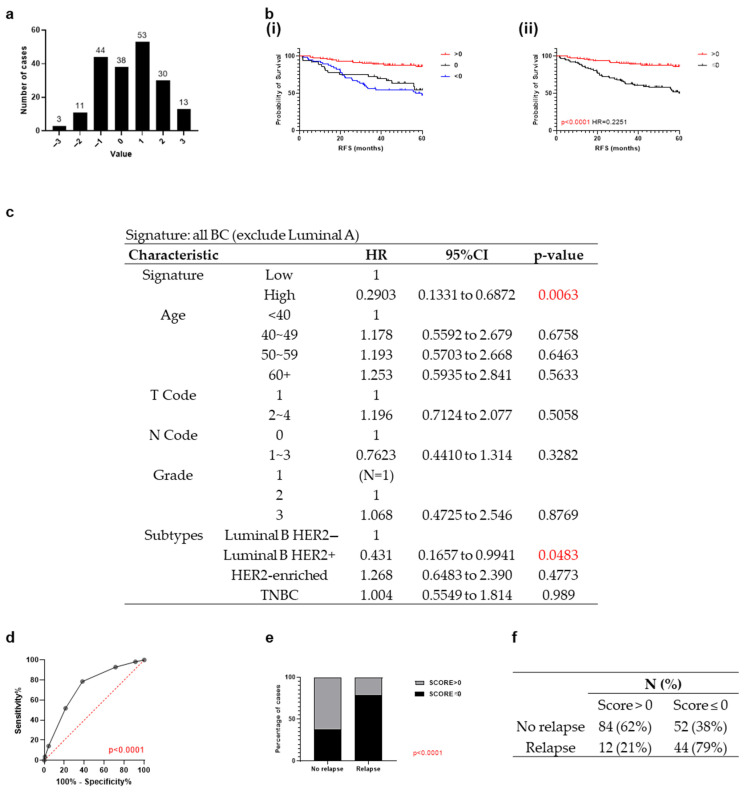
Frequency distribution of the five-biomarker signature score and the association between signature score and survival in the in-house Breast 300 cohort. (**a**) Graph representing the signature score for the five-biomarker signature in the Breast 300 cohort (*N* = 292). (**b**) Kaplan–Meier curve of relapse-free survival of the Breast 300 cohort except Luminal A stratified based on the signature score of (**i**) >0 (*N* = 96), 0 (*N* = 38) and <0 (*N* = 58) and (**ii**) >0 (*N* = 96) and ≤ 0 (*N* = 96). (**c**) Multivariate Cox regression analysis on RFS of the five-biomarker signature in all breast cancer except Luminal A in the Breast 300 cohort. (**d**) ROC curve analysis (Wison/Brown method) showing the sensitivity, specificity and 95% confidence interval for breast cancer as a whole except Luminal A across a series of thresholds of signature score in the in-house Breast 300 cohort. The red cross labelled the sensitivity and specificity when the cutoff was 0. (**e**) Stacked bar graph with Fisher’s exact test showing breast cancer as a whole except Luminal A patient outcome with percentage of cases under the signature score of higher or no more than 0. (**f**) Summary of number of patients (*N*) and percentage (%) for no relapse or relapse under signature higher or lower than 0 within a follow-up duration of 5 years in the in-house Breast 300 cohort.

Of note, when investigating each biomarker individually, no biomarker was associated with outcome within the Luminal A subgroup (Table 5). Therefore, these patients (*N* = 79) were excluded from the combined analysis. When applied to the remaining 192 relevant cases, a signature score ranging from −3 to 3 was observed with most of the patients having a signature score of 1, followed by −1, 0 and 2. Less than 10% of patients had a signature score of 3, −2 and −3 (Figure 3a).

To investigate the relationship between the signature score and patient outcome, patients were first stratified into three groups, signature > 0, 0 and <0 (*N* = 96, 38, 58, respectively) (Figure 3b(i)). Patients with a signature score > 0 were associated with better RFS than patients with a signature score = 0 and <0 (signature score > 0 vs. =0: HR = 0.2643, *p* = 0.0002; signature score > 0 vs. <0: HR = 0.2060, *p* < 0.0001). As the two groups, signature = 0 and <0, were closely aligned, they were combined as one group (Figure 3b(ii)). Patients with a signature score higher than 0 were approximately five times less likely to relapse compared to those with a score ≤ 0 (HR = 0.2251, *p* < 0.0001). Multivariate Cox regression analysis provided further evidence that this five-biomarker signature, within all breast cancer subtypes except Luminal A, may serve as an independent prognostic signature (Figure 3c).

The signature score stratification was then further investigated by directly correlating to patient outcome regardless of the relapse time. The ROC curve analysis showed statistical significance (*p* < 0.0001) with an area under the curve (AUC) of 0.7302 (Figure 3d). The signature score achieved a sensitivity of 79% who remained relapse-free and a specificity of 62% for identifying those who relapse (Figure 3e,f).

### 3.4. Gene Expression of the Five Biomarkers Cannot Be a Surrogate for Protein Expression

There are limited protein-based publicly available datasets, especially those based on IHC. However, as gene expression was also available for the same patients represented by the TMA [16], this raised the possibility of migrating the protein-based assay to gene expression so as to allow validation in the numerous publicly available gene expression datasets. Therefore, protein expression of the five biomarkers was correlated with the corresponding gene expression in the in-house Breast 300 cohort [16]. However, the analysis indicated that for all five biomarkers, only weak to moderate correlations were observed between protein and gene expression (Table 6).

To further investigate this relationship, protein–gene correlations were examined in publicly available datasets, including TCGA Firehose Legacy and CPTAC Cell 2020 [29]. Consistent with the in-house findings, weak correlations were generally observed when comparing microarray- or RNA-seq-based gene expression with RPPA-derived protein levels in TCGA. However, in CPTAC cohorts where protein abundance was quantified by mass spectrometry, stronger correlations were observed for selected biomarkers, suggesting that assay methodology may influence concordance between transcript and protein levels (Table S7).

Therefore, it was concluded gene expression could not be used as a reliable surrogate for protein expression for these biomarkers and alternate approaches would be required to further evaluate the signature performance.

### 3.5. The Five-Biomarker Signature Provided Superior Prognostic Value Compared to Published Chemotherapy-Associated Signatures

**Figure 4:**
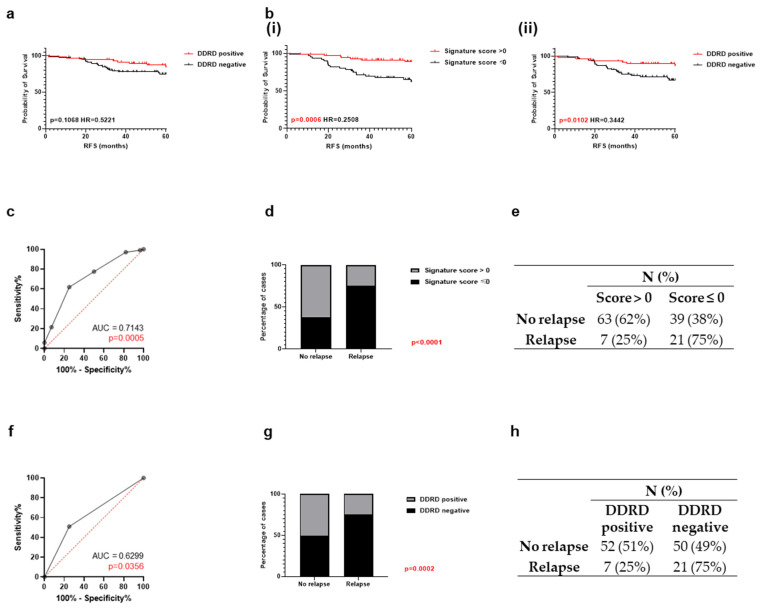
Association between DDRD signature score and survival in the in-house Breast 300 cohort compared to the five-biomarker signature. Kaplan–Meier curve of RFS of the Breast 300 cohort (**a**) as a whole stratified based on DDRD signature (DDRD positive: *N* = 59; DDRD negative: *N* = 98) and (**b**) except Luminal A stratified based on (**i**) five-biomarker signature (Signature score > 0: *N* = 66; Signature score ≤ 0: *N* = 45) and (**ii**) DDRD signature (DDRD positive: *N* = 51; DDRD negative: *N* = 60). ROC curve analysis (Wison/Brown method) showing the sensitivity, specificity and 95% confidence interval for breast cancer as a whole except Luminal A across a series of thresholds of (**c**) five-biomarker and (**f**) DDRD signature score in the in-house Breast 300 cohort. The red cross labelled the sensitivity and specificity when it was the chosen cutoff. Stacked bar graph with Fisher’s exact test showing breast cancer as a whole except Luminal A patient outcome with percentage of cases under the (**d**) five-biomarker and (**g**) DDRD signature score. Summary of number of patients (*N*) and percentage (%) for no relapse or relapse under (**e**) five-biomarker and (**h**) DDRD signature score within a follow-up duration of 5 years in the in-house Breast 300 cohort.

While it was not possible to migrate the IHC-based signature to a gene expression-based approach, the availability of the matched gene expression did allow comparison of the five-biomarker IHC signature to published gene expression-based prognostic breast cancer signatures in the same patient cohort. Review of the literature revealed multiple signatures, but most were precluded from comparison for a variety of reasons including lack of missing information (e.g., weighting) to allow replication and specific genes from a given signature not represented on the microarray (Table S8). Many signatures were also restricted to specific subgroups of BC rather than BC in general (except Luminal A). Only one signature however was available for comparison. The DNA damage response deficiency (DDRD) signature developed by Mulligan et al. [16] identifies tumours with impaired Fanconi anaemia/BRCA (FA/BRCA) pathway function, which are characterised by increased sensitivity to DNA-damaging agents. In the original study, DDRD-positive tumours were associated with improved relapse-free survival (HR = 0.37, *p* = 0.03). Application of this signature to the in-house Breast 300 cohort (*N* = 111, including only patients with both DDRD and five-biomarker scores) showed that DDRD-positive patients had better outcomes, although the difference did not reach statistical significance (HR = 0.52, *p* = 0.11). In contrast, the five-biomarker signature score > 0 identified patients approximately four times less likely to relapse (HR = 0.25, *p* = 0.0006) in all breast cancers, excluding Luminal A. The five-biomarker signature also demonstrated a higher discriminatory ability (AUC = 0.71, *p* = 0.0005) compared with the DDRD signature (AUC = 0.63, *p* = 0.036). It correctly classified 62% of non-relapsed and 75% of relapsed cases, outperforming the DDRD signature, which identified 51% and 75%, respectively (Figure 4).

**Figure 5:**
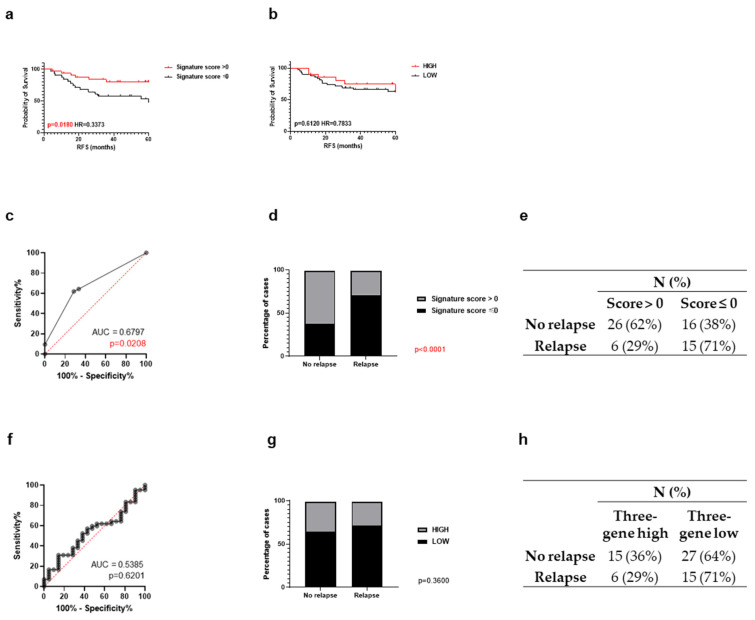
Association between three-gene signature score and survival in the in-house Breast 300 cohort compared to the five-biomarker signature. Kaplan–Meier curve of RFS of the Breast 300 cohort in TNBC stratified based on (**a**) five-biomarker signature (Signature score > 0: *N* = 32; Signature score ≤ 0: *N* = 31) and (**b**) three-gene signature (High: *N* = 21; Low: *N* = 42). ROC curve analysis (Wison/Brown method) showing the sensitivity, specificity and 95% confidence interval for TNBC across a series of thresholds of (**c**) five-biomarker and (**f**) three-gene signature score in the in-house Breast 300 cohort. Stacked bar graph with Fisher’s exact test showing TNBC patient outcome with percentage of cases under the (**d**) five-biomarker and (**g**) three-gene signature score. Summary of number of patients (*N*) and percentage (%) for survival or relapse under (**e**) five-biomarker and (**h**) three-gene score within a follow-up duration of 5 years in the in-house Breast 300 cohort.

Given the limited number of suitable signatures available for direct comparison with the five-biomarker model in the adjuvant setting, the scope of comparison was expanded to include signatures developed in the neoadjuvant chemotherapy context. Accordingly, a second comparison was made with the three-gene E2F target signature (CDKN2C, DEK, MCM3) established by Oshi et al. [30], which predicts response to neoadjuvant chemotherapy in TNBC. This comparison was undertaken with the explicit acknowledgement that the E2F signature was originally derived in the neoadjuvant setting and is here applied to an adjuvant-treated cohort as an exploratory benchmark rather than a direct clinical analogue. In the Breast 300 TNBC subset (*N* = 63), a five-biomarker signature score > 0 was associated with a markedly reduced relapse risk (HR = 0.34, *p* = 0.018), whereas the three-gene signature showed no significant prognostic value (HR = 0.78, *p* = 0.61). Similarly, the five-biomarker signature achieved a higher AUC (0.68, *p* = 0.021) than the three-gene signature (0.54, *p* = 0.62) and better classified non-relapsed cases (62% vs. 36%), while performance for relapsed cases was similar (71% vs. 71%; Figure 5). Together, these comparisons indicate that the five-biomarker signature provides superior discriminatory power compared with the DDRD and three-gene signatures in chemotherapy-treated breast cancer, particularly in non-Luminal A and TNBC subtypes.

## 4. Discussion

In this study, we developed a novel five-biomarker signature for predicting the patient outcome in the context of adjuvant anthracycline-based chemotherapy in breast cancer. Five hypothesis-driven biomarkers, TOP2A, PTEN, EGFR, IGF1R, and p-mTOR, were robustly quantified using digital pathology and individually demonstrated significant associations with improved RFS in specific breast cancer subtypes. When combined into a composite five-biomarker signature, patients with a high signature score exhibited significantly better outcomes than those with a low signature score. This finding indicates that the five-biomarker signature may serve as a useful prognostic tool for predicting patient outcomes in the context of anthracycline-based chemotherapy. Furthermore, as each biomarker represents a potentially actionable molecular target, this signature may not only predict patient outcome in the context of chemotherapy treatment but also help guide the selection of alternative targeted therapies for patients unlikely to benefit from standard regimens.

A key strength of this study lies in the use of digital pathology for biomarker quantification. Unlike labour-intensive and subjective manual IHC scoring, which relies on discrete ordinal scales, the use of the digital pathology platform, QuPath, enables semi-automated analysis of whole-TMA images, delivering continuous measurements that integrate staining intensity with cellular and subcellular morphology [31]. Compared with other digital pathology platforms, QuPath can distinguish the subcellular compartments, enhancing biological interpretability. For example, in the study by Matos et al. which used a digital software, ImageLab (Softium Informática, São Paulo, Brazil), to quantify the staining of Galectin-3 in 25 cases of thyroid carcinoma [32], only a moderate correlation for overall expression was obtained when compared to manual quantification (Pearson correlation coefficient, r = 0.66, *p* = 0.0001) [32]. This may be underpinned by the fact that ImageLab determines positivity by marking cells with binary dots, without accounting for the biological characteristics of individual cells. This lack of contextual cellular information may explain the weaker correlation observed for overall expression. In contrast, QuPath, as applied in this study, incorporates cellular morphology by first identifying the nucleus based on a user-defined threshold and then expanding a proper area as cytoplasmic regions, also customisable by the user. The open-source nature and built-in machine-learning capabilities of QuPath further distinguish it from costly commercial AI platforms (e.g., Paige AI, which is FDA-cleared for prostate cancer but restricted in scope and affordability [33]. Although QuPath requires greater user oversight to maintain data quality, its adaptability and accessibility make it an invaluable tool for biomarker research and digital pathology development. If further validated, the algorithms developed through QuPath-based quantification could be adapted for automated clinical assessment of the same biomarkers.

To investigate the robustness of the five-biomarker signature, we explored if it was possible to translate it into a gene-based signature and apply it to publicly available datasets for comparison with existing prognostic signatures. However, no strong correlation was observed between protein and gene expression for the five biomarkers, consistent with previous reports. For example, Anita et al. observed only a moderate correlation between TOP2A protein expression and gene amplification [34]. Similarly, PTEN protein loss has been shown to occur independently of mRNA expression [35]. IGF1R have also demonstrated weak concordance between mRNA and protein levels in breast cancer and other tumour types [36,37]. In the case of mTOR, pathway activation is primarily governed by phosphorylation status rather than gene expression, and phospho-mTOR levels show little correlation with mTOR transcript abundance [38]. As the gene expression data was generated using a microarray, there is the potential that this could be due to the relevant probeset(s) not accurately reflecting the expression of the gene. However, a small in-house dataset where the same microarray was used to analyse a panel of 15 cell lines was used to validate the probesets comparing the findings to those reported in RNA-Seq-based studies for the same cell lines or in-house qPCR data. Instead, the weak correlation may reflect the multiple levels of regulation between mRNA transcription and final protein abundance. After transcription, pre-mRNA undergoes post-transcriptional modifications such as alternative splicing and 3′ end processing, meaning not all transcripts become mature, translatable mRNA [39]. Moreover, total RNA extraction includes various RNA species, and methodological differences can influence quantification outcomes [40]. Translation efficiency is also gene dependent, influenced by codon usage, ribosome availability, and regulatory interactions [41]. Additionally, post-translational modifications such as phosphorylation and ubiquitination further affect protein stability, localisation, and activity independently of mRNA levels [42,43].

The Luminal A subtype was not included in the combined five-biomarker signature, as none of the biomarker-defined stratification approaches captured this group. This exclusion is therefore inherent to the biological scope of the selected markers rather than a result of selective analysis. Although this introduces subtype specificity, its impact may be limited as Luminal A is generally associated with a more favourable prognosis and lower risk of relapse compared to other subtypes. Nevertheless, this limitation should be acknowledged. Combining multiple biomarkers into a single composite score improved prognostic accuracy relative to individual markers, mitigating the limitations of sensitivity or specificity inherent to single biomarkers. The five-biomarker signature achieved a sensitivity of 62% and a specificity of 79%, indicating a strong ability to identify patients less likely to benefit from anthracycline therapy. Although the moderate sensitivity suggests some high-risk cases may remain unrecognised, the relatively high specificity enhances its clinical usefulness in identifying patients who may require additional or alternative therapies early in their treatment course. Beyond statistical performance, the use of protein-based biomarkers offers translational advantages over gene-based assays. As previously mentioned, protein expression more directly reflects functional tumour biology, capturing post-transcriptional regulation and post-translational modifications not evident at the mRNA level. IHC, the platform underpinning this signature, is already a standard method in pathology laboratories, making implementation straightforward. In contrast, gene expression assays often require specialised facilities and are more costly and time-consuming. The reduced complexity and cost of IHC-based assessment therefore enhance the practicality of this approach for integration into routine clinical workflows.

To evaluate the robustness of the five-biomarker signature, published gene expression-based signatures were applied to the Breast 300 cohort for comparison. The DDRD signature met the necessary criteria (signature for whole breast cancer and treated with adjuvant anthracycline-based chemotherapy) and was selected for comparison. The DDRD signature, developed in overlapping patient cohorts, showed a weaker association with survival when reanalysed, likely due to the exclusion of Luminal A cases, which generally have better prognosis. Despite this, the five-biomarker signature retained its prognostic significance, suggesting improved robustness and applicability. Unlike the DDRD signature, which relies on 44 genes, the five-biomarker model’s simplicity enhances its feasibility and cost-effectiveness for clinical implementation [16]. The three-gene signature [30], developed from neoadjuvant-treated cohorts, failed to show significance in the Breast 300 cohort, likely reflecting differences in experimental context (adjuvant vs. neoadjuvant) and microarray platform (Almac Breast DSA vs. U133A). In the adjuvant context, the removal of the primary tumour and the resulting alterations in the tumour microenvironment could attenuate the predictive relevance of signatures derived for neoadjuvant studies. Additionally, the restriction of the three-gene signature to TNBC limits its clinical reach compared with the broader applicability of the five-biomarker model, which effectively stratified all subtypes except Luminal A.

The five biomarkers have been widely studied for their prognostic and therapeutic relevance in breast cancer. Beyond their association with patient outcomes, each represents a potentially actionable target, suggesting that patients predicted to have poor responses to chemotherapy might benefit from alternative targeted therapies based on their biomarker profile. However, it is critical to determine whether the diagnostic thresholds used to define high or low expression align with the clinical cut-offs required for therapeutic intervention. Furthermore, for patients exhibiting high biomarker expression, outcomes might be further optimised by integrating these targeted agents with standard chemotherapy regimens. TOP2A encodes topoisomerase IIα, the molecular target of anthracyclines such as epirubicin, which was used in the Breast 300 cohort [44]. Consistent with this, patients with high TOP2A expression demonstrated improved outcomes, supporting its predictive value for anthracycline sensitivity. PTEN, a tumour suppressor regulating cell proliferation and survival, has been linked to increased sensitivity to PARP inhibitors [45]. Preclinical studies have shown that PTEN-deficient cells are substantially more responsive to agents such as olaparib, highlighting potential therapeutic avenues for patients with PTEN loss [46,47,48]. EGFR can be targeted by monoclonal antibodies (e.g., cetuximab) and tyrosine kinase inhibitors (e.g., lapatinib, neratinib), several of which have shown efficacy in specific breast cancer subtypes [49,50,51,52,53,54]. Although IGF1R emerged early as a promising drug target, clinical translation remains limited due to inconsistent trial results [55]. Finally, mTOR inhibitors such as temsirolimus and everolimus are clinically approved agents that suppress cell cycle progression and have demonstrated benefit in hormone receptor-positive, HER2-negative breast cancer [56,57].

A limitation of the five-biomarker signature lies in its construction using an unweighted scoring system, where each biomarker contributes equally to the overall score. While this unweighted approach provides a transparent and easily interpretable model, it assumes equivalent prognostic contribution across markers. A statistically weighted system, for example, based on hazard ratios derived from Cox regression, may further refine its prognostic accuracy. Moreover, a key limitation of this study is the potential for overfitting, as the five-biomarker signature was both developed and assessed within the same cohort, without the use of internal validation approaches such as resampling. This may lead to an overestimation of its prognostic performance. Furthermore, the protein-based nature of the signature limited the availability of comparable external datasets, and therefore independent validation could not be performed. The absence of both internal and external validation limits the assessment of model robustness and may increase the risk of Type I and Type II errors. Additionally, the retrospective design of the Breast 300 cohort may introduce inherent biases. Although all patients received anthracycline-based chemotherapy, which provides a degree of treatment uniformity and reduces variability in therapeutic exposure, this may also limit the generalisability of the findings to patients receiving alternative regimens. In the meantime, although the five-biomarker signature was developed within this chemotherapy-treated cohort, it currently serves as a prognostic rather than predictive marker, as all patients received standard of care chemotherapy. This limitation, common to most prognostic models developed in similar settings, underscores the need for future validation in independent, treatment-stratified cohorts [30,58,59,60]. Finally, to be acknowledged, the absence of supporting biological or functional experiments and the observational nature of this study restrict the ability to draw causal conclusions, and the findings should therefore be interpreted as associative rather than indicative of direct biological effects. Therefore, future work should focus on validating these findings in larger, well-characterised independent cohorts with available protein expression data. Where feasible, prospective studies or controlled clinical trials would provide more robust evidence of clinical utility. Incorporation of internal validation approaches, such as resampling strategies, may also help to assess the stability of the model.

## 5. Conclusions

To conclude, this study established a protein-based five-biomarker signature capable of predicting patient outcome in the context of standard anthracycline-based chemotherapy across multiple breast cancer subtypes, excluding Luminal A. Although the exclusion of Luminal A is notable, it is less critical given the generally favourable prognosis of this subtype. Unlike most existing signatures that focus on distinguishing low- from high-realpse risk patients to guide chemotherapy administration, this signature specifically addresses the prognostic stratification of high-risk patients already receiving chemotherapy, offering potential value in guiding alternative or supplementary treatment strategies. Its protein-based nature confers practical advantages, reflecting the functional state of tumour biology and allowing straightforward implementation through standard immunohistochemistry. Nonetheless, the limited availability of large protein-based datasets and the need for standardised IHC quantification remain challenges for broader validation. Future research should focus on expanding the range of biomarkers and validating the signature in larger proteomic cohorts, which may enhance its prognostic precision and enable its application across all breast cancer subtypes, including Luminal A.

## Figures and Tables

**Table 1:** Summary of clinical information including patient number (*N*) and event number (*n*) for breast cancer TMA of Breast 300.

Breast 300 (*N* = 293)		
**Tumour grade**	***N***	**%**
G1	4	1
G2	114	39
G3	153	52
Unknown	22	8
**Histological type**	***N***	**%**
Ductal (no special type)	15	5
Invasive ductal	195	67
Invasive lobular	31	11
Mixed ductal and lobular	25	9
Other	7	2
Unknown	21	7
**Therapies**	***N***	**%**
Radiotherapy	228	78
Herceptin	48	16
Hormone therapy	167	57
**St Galen Subtypes**	***N* (*n*)**	**% (*n*%)**
Luminal A	79 (10)	27 (13)
Luminal B HER2−	64 (14)	22 (22)
Luminal B HER2+	33 (10)	11 (30)
HER2-enriched	39 (13)	13 (33)
TNBC	72 (22)	25 (31)
Unknown	6 (0)	2 (0)

**Table 2:** Summary of the specific clone, optimised antibody conditions and automated platform utilised for immunohistochemical analysis.

Antibody	Clone	Dilution/Pre-Treatment	Company	Automated Platform
TOP2A	JS5B4	Pre-set/CC1 mild	Ventana	Ventana DISCOVERY XT
EGFR	3C6	Pre-set/Protease 12 min	Ventana	Ventana DISCOVERY XT
IGF1R	G11	Pre-set/CC1 mild	Ventana	Ventana DISCOVERY XT
PTEN	6H2.1	1:1600/ER2 20 min	Dako	Leica BOND-MAX
p-mTOR	49F9	1:100/ER2 20 min	Cell Signalling	Leica BOND-MAX
ER	6F11	1:200/ER2 20 min	Leica	Leica BOND-MAX
PR	PgR 636	1:150/ER1 20 min	Dako	Leica BOND-MAX
HER2	CB11	Pre-set/ER1 25 min	Leica	Leica BOND-MAX
Ki67	MM1	1:200/ER2 30 min	Leica	Leica BOND-MAX

**Table 3:** Summary of the subcellular staining position and scoring method of the five biomarkers.

Biomarker	Localisation	Scoring Method
TOP2A	tumour nucleus	Percentage of positive tumour cells
PTEN	tumour nucleus	H-score
EGFR	whole tumour cell	H-score
IGF1R	whole tumour cell	H-score
p-mTOR	whole tumour cell	H-score
	peri-nuclear region	Percentage of positive tumour cells

**Table 4:** Summary of the expression position, cutoff used in specific subtypes of the five biomarkers showed association with clinical outcome, and the associated *p*-value and HR value.

Biomarker	Localisation	Subtype	Threshold	*p* Value	HR
TOP2A	tumour nucleus	ER−	45%	0.003	0.287
PTEN	tumour nucleus	HER2+ and Luminal B HER2−	100 (median value)	0.0037	0.356
EGFR	whole tumour cell	All subtypes except Luminal A	extremely strong staining	0.203	0.2979
IGF1R	whole tumour cell	Luminal B HER2−	219 (median value)	0.0419	0.2816
p-mTOR	whole tumour cell	Luminal B	100	0.0225	0.3832
	peri-nucleus	HER2+	1%	0.006	0.2977

**Table 5:** Summary of the breast cancer subtypes that were captured by the five-biomarker signature. The captured subtypes were ticked.

Biomarker	Localisation	Luminal A	Luminal B HER2−	Luminal B HER2+	HER2-Enriched	TNBC
TOP2A	tumour nucleus				√	√
PTEN	tumour nucleus		√	√	√	
EGFR	whole tumour cell		√	√	√	√
IGF1R	whole tumour cell		√			
p-mTOR	whole tumour cell		√	√		
	peri-nucleus			√	√	

**Table 6:** Summary of the protein expression position of the five biomarkers and their correlation with the gene expression.

Biomarker	Position	Correlation Between Protein and Gene	
TOP2A	tumour nucleus	*p* < 0.0001, r = 0.6559	moderate
PTEN	tumour nucleus	*p* < 0.0001, r = 0.3968	weak
EGFR	whole tumour cell	*p* = 0.0594, r = 0.1153	weak
IGF1R	whole tumour cell	*p* < 0.0001, r = 0.6907	moderate
p-mTOR	whole tumour cell	*p* = 0.0097, r = 0.1663	weak
	peri-nucleus	*p* = 0.1412, r = 0.0903	weak

## Data Availability

The gene expression data matched to the Breast 300 cohort in have previously been published [16]. No new gene expression data were generated. Protein immunohistochemistry scores generated for the current study are not publicly available but may be obtained from the corresponding author upon reasonable request, subject to ethical and data governance considerations.

## Supplementary Materials

The following supporting information can be downloaded at: https://www.mdpi.com/article/10.3390/cancers18071092/s1, Figure S1: IHC score of the five biomarkers; Figure S2: Association between p-mTOR protein expression and survival in the in-house Breast 300 cohort; Figure S3: Association between p-mTOR protein expression in peri-nucleus and survival in the in-house Breast 300 cohort; Table S1: Correlation between TOP2A expression and survival; Table S2: Correlation between PTEN expression and survival; Table S3: Correlation between EGFR expression and survival; Table S4: Patients with extremely strong EGFR staining; Table S5: Correlation between IGF1R expression and survival; Table S6: Multivariate Cox regression analysis on RFS of five biomarkers; Table S7: Summary of the correlation between protein and gene expression in the TCGA Firehose Legacy and CPTAC cell 2020 datasets; Table S8: Summary of signatures for predicting the patient outcome in breast cancer in the context of chemotherapy treatment and the reason they were not applicable to the Breast 300. References [58,59,60,61,62,63,64] are cited in the Supplementary Materials.

## Abbreviations

The following abbreviations are used in this manuscript:

SoC	Standard of care
ER	Oestrogen receptor
PR	Progesterone receptor
HER2	Human epidermal growth factor receptor 2
pCR	Pathological complete response
TOP2A	Topoisomerase II alpha
PTEN	Phosphatase and TENsin Homologue
EGFR	Epidermal Growth Factor Receptor
IGF1R	Insulin-like Growth Factor 1 Receptor
mTOR	Mammalian Target of Rapamycin
PNL	Purely non-invasive lesions
CEIN	Co-existing invasive cancer
DCIS	Ductal carcinoma in situ
TMA	Tissue microarray
TNBC	Triple negative breast cancer
IHC	Immunohistochemistry
N	Patient number
n	Event number
FFPE	Formalin-fixed paraffin-embedded
H-score	Histochemical score
AUC	Area under the curve
DDRD	DNA damage response deficiency
FA/BRCA	Fanconi anaemia/BRCA
WSIs	Whole-slide images
AI	Artificial intelligence
