# Supplementary material for: A Five-Biomarker IHC-Based Signature Predicting Outcome in Breast Cancer Patients Following Adjuvant Anthracycline-Based Chemotherapy
Source: Cancers (Basel). 2026 Mar 27;18(7):1092. doi: 10.3390/cancers18071092 (PMC13071981; doi:10.3390/cancers18071092)
Supplement: Supplementary file 1 [file cancers-18-01092-s001.zip › cancers-4201204-supplementary.pdf]

Figure S1. IHC score of the five biomarkers

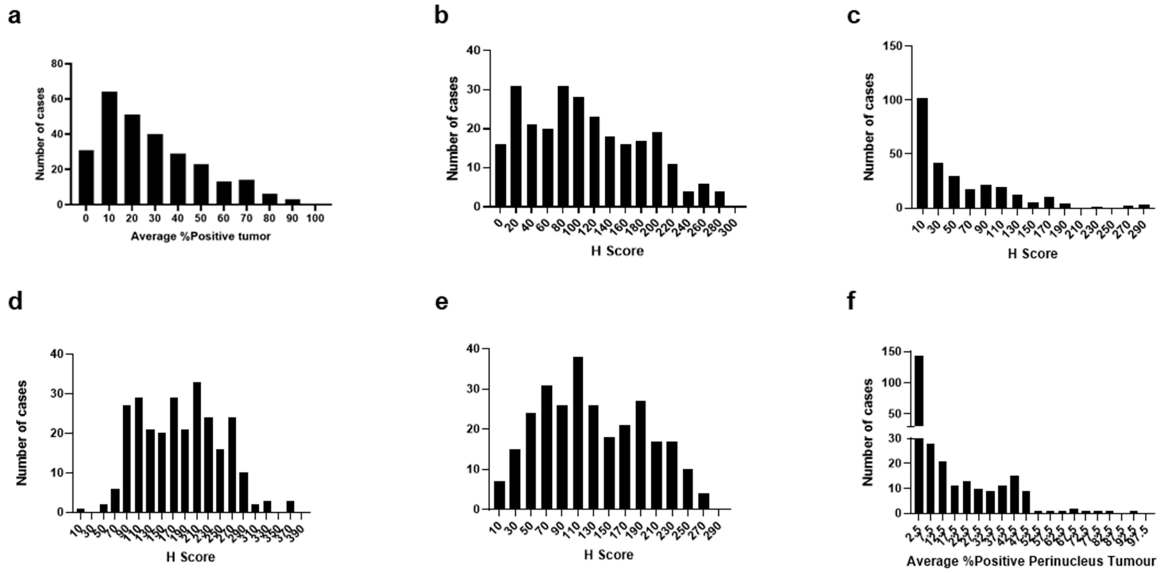

Frequency distribution showing protein expression of **(a)** TOP2A in tumour nucleus (N=273), **(b)** PTEN in tumour nucleus (N=265), **(c)** IGF1R in tumour cells (N=268), **(d)** EGFR in tumour cells (N=271), **(e)** p-mTOR in tumour cells (N=281) and **(f)** p-mTOR in peri-nucleus (N=281).

Table S1. Correlation between TOP2A expression and survival.

Summary of the number of patients (N), log-rank p-value and hazard ratio (HR) for survival analysis of relapse-free survival with follow-up duration of 5 years stratified by the cutoff of TOP2A in the in-house Breast 300 cohort.

Subtypes (RFS)	N	cutoff	p value	HR
All breast cancer	293	0.23	0.0068	2.019
All breast cancer (OS)	293	0.23	0.0731	1.747
Stratified by the median value of the entire cohort				
Luminal A	75	23%	0.1272	3.18
Luminal B HER2-	59	23%	0.2364	2.033
Luminal B HER2+	28	23%	0.2431	2.533
HER2-enriched	38	23%	0.6473	0.7677
TNBC	71	23%	0.8029	0.8713
Stratified by the median value of each subtype				
Luminal A	75	8%	0.7107	1.282
Luminal B HER2-	59	24%	0.1442	2.377
Luminal B HER2+	28	24%	0.1568	3.026
HER2-enriched	38	26%	0.4491	0.6463
TNBC	71	45%	0.0091	0.3081
Stratified by the median value of TNBC				
Luminal A	75	45%	0.7143	0.3624[1]
Luminal B HER2-	59	45%	0.3056	1.954
Luminal B HER2+	28	45%	0.5573	0.5414
HER2-enriched	38	45%	0.1276	0.3020[#]
ER-	109	45%	0.003	0.287
Stratified by the Cutoff Finder of each subtype				
ER-negative	109	44.70%	0.003	0.287
TNBC	71	41.97%	0.0048	0.2975
HER2-enriched	38	29.11%	0.0532	0.2523

[1] For the patient group without any observed events, the Mantel-Haenszel test was utilised.

Table S2. Correlation between PTEN expression and survival.

Summary of the log-rank p-value, hazard ratio 95% CI of ratio and number of patients (N) and events (n) for survival analysis of relapse-free survival with follow-up duration of 5 years stratified by the cutoff of PTEN in the in-house Breast 300 cohort.

Subtypes (RFS)	N	cutoff	p value	HR
All breast cancer	265	100	0.0034	0.4679
All breast cancer (OS)	265	100	0.0517	0.5376
**Stratified by the median value of the entire cohort**				
Luminal A	74	100	0.3974	0.5712
Luminal B HER2-	55	100	0.0454	0.3376
Luminal B HER2+	28	100	0.3752	0.5401
HER2-enriched	35	100	0.1	0.3076
TNBC	71	100	0.9791	0.9876
**Stratified by the median value of each subtype**				
Luminal A	74	110	0.8626	0.5246
Luminal B HER2-	55	132	0.1646	0.4441
Luminal B HER2+	28	120	0.383	0.5346
HER2-enriched	35	72	0.0463	0.293
TNBC	71	66	0.9127	0.9543
HER2+ and Luminal B HER2-	118	100	0.0037	0.356
**Stratified by the Cutoff Finder of each subtype**				
Luminal B HER2-	55	78.1	0.014	0.28
Luminal B HER2+	28	139.7	0.082	0.19
HER2-enriched	35	76.91	0.017	0.19
HER2+ and Luminal B HER2-	118	83.05	0.0013	0.45

Table S3. Correlation between EGFR expression and survival.

Summary of the log-rank p-value, hazard ratio 95% CI of ratio and number of patients (N) and events (n) for survival analysis of relapse-free survival with follow-up duration of 5 years stratified by the cutoff of EGFR in the in-house Breast 300 cohort.

Subtypes (RFS)	N	cutoff	p value	HR
All breast cancer	268	28.8	0.4581	1.2
All breast cancer (OS)	268	28.8	0.0922	1.674
**Stratified by the median value of the entire cohort**				
Luminal A	77	28.8	0.7212	0.7947
Luminal B HER2-	61	28.8	0.8644	0.9025
Luminal B HER2+	30	28.8	0.2261	0.299
HER2-enriched	35	28.8	0.7737	1.188
TNBC	63	28.8	0.7749	1.172
**Stratified by the median value of each subtype**				
Luminal A	77	22.7	0.5307	0.669
Luminal B HER2-	61	16.7	0.9025	1.07
Luminal B HER2+	30	8.7	0.2304	0.4388
HER2-enriched	35	53.5	0.0702	2.81
TNBC	63	95.8	0.7852	0.888
**Stratified by the Cutoff Finder of each subtype**				
All breast cancer	268	0.8	0.0017	0.3063
Luminal A	75	5	0.0648	0.3829[1]
Luminal B HER2-	59	7.6	0.2426	0.5286
Luminal B HER2+	28	13.9	0.2042	0.3769
HER2-enriched	38	95.5	0.0816	2.622
TNBC	109	122	0.2595	1.631

[1] For the patient group without any observed events, the Mantel-Haenszel test was utilised.

Table S4. Eleven patients with extremely strong EGFR staining and their survival.
Summary of patient ID, EGFR expression (H-score), St Galen subtypes, relapse-free survival time and events in the patients with extremely high EGFR staining in the in-house Breast 300 cohort.

Patient ID	H Score	GALEN	RFS (months)	Event
P03-11690	297.9	TNBC	5.901639	1
P03-05705	289.38	TNBC	60	0
P08-00354	288.93	TNBC	42.95082	0
P08-22530	276.48	HER2-enriched	16.1753	0
P03-07438	273.06	Luminal A HER2-	60	0
P05-07296	238.93	HER2-enriched	60	0
PP00-13217	195.36	TNBC	60	0
PP00-15431	192.77	Luminal A HER2-	55.80328	0
P08-06001	172.06	TNBC	37.67213	0
P08-04436	170.32	TNBC	42	0
P08-05576	167.05	HER2-enriched	38.45902	0

Table S5. Correlation between IGF1R expression and survival

Summary of the log-rank p-value, hazard ratio 95% CI of ratio and number of patients (N) and events (n) for survival analysis of relapse-free survival with follow-up duration of 5 years stratified by the cutoff of IGF1R in the in-house Breast 300 cohort.

Subtypes (RFS)	N	cutoff	p value	HR
All breast cancer	271	180	0.0382	0.5862
All breast cancer (OS)	271	180	0.0863	0.584
**Stratified by the median value of the entire cohort**				
Luminal A	74	180	0.3398	0.5327
Luminal B HER2-	59	180	0.1955	0.477
Luminal B HER2+	29	180	0.7761	0.8062
HER2-enriched	37	180	0.561	0.3555[1]
TNBC	70	180	0.4098	1.426
**Stratified by the median value of each subtype**				
Luminal A	74	202.7	0.767	0.8201
Luminal B HER2-	59	219	0.0419	0.2816
Luminal B HER2+	29	165	0.6932	1.347
HER2-enriched	37	101	0.9605	1.029
TNBC	70	150.7	0.7596	0.8774
**Stratified by the Cutoff Finder of each subtype**				
All breast cancer	74	268.6	0.052	3.61
Luminal A	59	208.1	0.0157	0.23
Luminal B HER2-	29	154	0.4098	1.956
Luminal B HER2+	37	95	0.1792	2.707
HER2-enriched	70	240.9	0.0575	2.525

[1] For the patient group without any observed events, the Mantel-Haenszel test was utilised.

Figure S2. Association between p-mTOR protein expression and survival in the in-house Breast 300 cohort. (a) Summary of the number of patients (N), log-rank p-value and hazard ratio (HR) for survival analysis of relapse-free survival with follow-up duration of 5 years. Kaplan Meier curve of relapse-free survival within St. Gallen Subtypes Luminal B based on (b) tertiles and (c) the high (above 100) and low (below 100) p-mTOR whole-cell protein expression with follow-up duration of 5 years in the in-house Breast 300 cohort.

a

Subtypes (RFS)	N	cutoff	p value	HR
All breast cancer	279	119.8	0.5834	0.8736
All breast cancer (OS)	279	119.8	0.9628	0.9861
**Stratified by the median value of the entire cohort**				
Luminal A	77	119.8	0.3154	2.169
Luminal B HER2-	61	119.8	0.327	0.5838
Luminal B HER2+	30	119.8	0.1986	0.4041
HER2-enriched	37	119.8	0.6182	1.354
TNBC	72	119.8	0.2109	1.794
**Stratified by the median value of each subtype**				
Luminal A	77	170	0.1579	2.461
Luminal B HER2-	61	141	0.6556	0.781
Luminal B HER2+	30	128	0.0734	0.2591
HER2-enriched	37	127	0.499	1.48
TNBC	72	78.3	0.7101	0.8566
**Stratified by the Cutoff Finder of each subtype**				
Luminal A	77	188.1	0.0949	3.067
Luminal B HER2-	61	77.1	0.0844	0.3877
Luminal B HER2+	30	104.4	0.0335	0.2403
HER2-enriched	37	138	0.0822	2.761
TNBC	72	122.4	0.1508	2.041

b

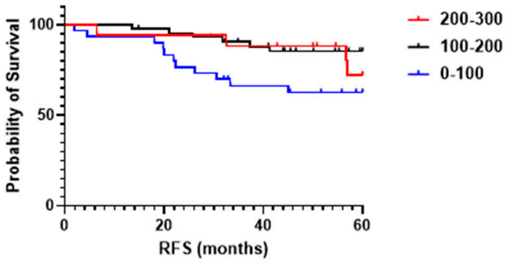

c

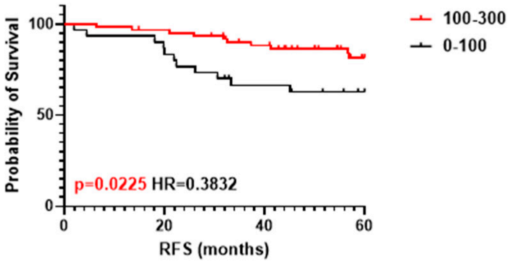

Figure S3. Association between p-mTOR protein expression in peri-nucleus and survival in the in-house Breast 300 cohort.
(a) Summary of the number of patients (N), log-rank p-value and hazard ratio (HR) for survival analysis of relapse-free survival with follow-up duration of 5 years. Kaplan Meier curve of relapse-free survival of the HER2-positive cases within the Breast 300 cohort stratified based on the **(b)** high (above 0.46%) and low (below 0.46%) or **(c)** high (above 1%) and low (below 1%) p-mTOR peri-nuclear protein expression.

a

Subtypes (RFS)	N	cutoff	p value	HR
All breast cancer	279	4.90%	0.1839	0.7194
All breast cancer (OS)	279	4.90%	0.4874	0.8067
**Stratified by the median value of the entire cohort**				
Luminal A	77	4.90%	0.6822	0.7683
Luminal B HER2-	61	4.90%	0.9547	0.9689
Luminal B HER2+	30	4.90%	0.3258	0.4958
HER2-enriched	37	4.90%	0.0621	0.3526
TNBC	72	4.90%	0.5443	0.7757
**Stratified by the median value of each subtype**				
Luminal A	77	4.06%	0.9783	1.017
Luminal B HER2-	61	3.58%	0.2463	0.4703
Luminal B HER2+	30	6.37%	0.8428	0.8957
HER2-enriched	37	7.80%	0.0693	0.3465
TNBC	72	4.17%	0.3949	0.701
**Stratified by the Cutoff Finder of each subtype**				
Luminal B HER2+	30	2.77%	0.0907	0.3118
HER2-enriched	37	3.52%	0.0036	0.2145
HER2+	72	0.46%	0.0001	0.2022
HER2+	72	1.00%	0.006	0.2977

b

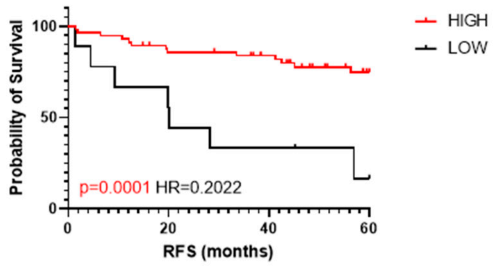

c

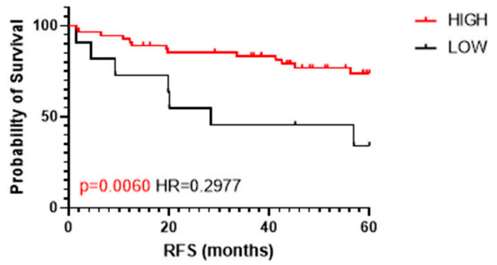

Table S6. Multivariate Cox regression analysis on RFS of five biomarkers

TOP2A: ER-

**Characteristic**		**HR**	**95%CI**	**p-value**
TOP2A	Low	1		
	High	0.3051	0.1114 to 0.7084	0.0046
Age	<40	1		
	40~49	0.3063	0.08887 to 1.053	0.0599
	50~59	0.6104	0.2216 to 1.883	0.3706
	60+	0.4382	0.1558 to 1.338	0.1405
T Code	1	1		
	2~4	0.6571	0.2873 to 1.607	0.3439
N Code	0	1		
	1~3	3.189	1.429 to 7.677	0.0042
Grade	2	1		
	3	0.7995	0.2895 to 2.859	0.7013

PTEN: HER2+ and Luminal B HER2-

**Characteristic**		**HR**	**95%CI**	**p-value**
PTEN	Low	1		
	High	51.39	17.70 to 189.6	<0.0001
Age	<40	1		
	40~49	0.6789	0.2872 to 1.721	0.3987
	50~59	0.5351	0.2198 to 1.381	0.1879
	60+	1.342	0.5456 to 3.503	0.5252
T Code	1	1		
	2~4	1.78	0.8962 to 3.862	0.1022
N Code	0	1		
	1~3	1.186	0.6393 to 2.286	0.5941
Grade	1	N=1		
	2	1		
	3	2.096	1.115 to 4.097	0.0212

EGFR: All subtypes except Luminal A

**Characteristic**		**HR**	**95%CI**	**p-value**
EGFR	Low	1		
	High	11.11	7.168 to 17.30	<0.0001
Age	<40	1		
	40~49	1.299	0.7703 to 2.246	0.3311
	50~59	1.393	0.8472 to 2.364	0.195
	60+	1.2	0.7358 to 2.016	0.4714
T Code	1	1		
	2~4	0.9238	0.6578 to 1.316	0.6551
N Code	0	1		
	1~3	0.9634	0.6929 to 1.342	0.8251
Grade	1	N=1		
	2	1		
	3	1.299	0.9225 to 1.849	0.1355

IGF1R: Luminal B HER2-

Characteristic		HR	95%CI	p-value
IGF1R	Low	1		
	High	19.93	5.673 to 73.98	<0.0001
Age	<40	1		
	40~49	0.3159	0.06912 to 1.307	0.1114
	50~59	0.4973	0.1647 to 1.576	0.2256
	60+	0.3434	0.1023 to 1.198	0.0916
T Code	1	1		
	2~4	0.5164	0.1995 to 1.398	0.1867
N Code	0	1		
	1~3	0.5153	0.1864 to 1.420	0.1982
Grade	1	N=1		
	2	1		
	3	1.183	0.4541 to 3.099	0.7293

p-mTOR whole cell: Luminal B

Characteristic		HR	95%CI	p-value
p-mTOR whole cell	Low	1		
	High	30.7	8.762 to 123.5	<0.0001
Age	<40	1		
	40~49	0.2998	0.07362 to 1.076	0.0647
	50~59	1.001	0.2885 to 3.520	0.999
	60+	0.5157	0.1817 to 1.542	0.228
T Code	1	1		
	2~4	2.572	0.8605 to 11.06	0.0957
N Code	0	1		
	1~3	0.3392	0.1343 to 0.8337	0.0184
Grade	1	N=1		
	2	1		
	3	1.172	0.4860 to 2.897	0.7253

p-mTOR peri-nucleus: HER2+

Characteristic		HR	95%CI	p-value
p-mTOR peri-nucleus	Low	1		
	High	20.56	3.953 to 162.3	0.0003
Age	<40	1		
	40~49	1.463	0.2086 to 12.64	0.6996
	50~59	0.6547	0.08993 to 5.543	0.6734
	60+	0.4915	0.06643 to 4.240	0.488
T Code	1	1		
	2~4	2.359	0.4978 to 18.27	0.2967
N Code	0	1		
	1~3	1.399	0.3263 to 7.552	0.6616
Grade	2			

3	0.6677	0.1748 to 3.018	0.5756

Table S7. Summary of the correlation between protein and gene expression in the TCGA Firehose Legacy and CPTAC cell 2020 datasets.

TCGA Firehose Legacy: correlation between RNA microarray and RPPA (N=403)

Biomarker	r	95% confidence interval	p value	correlation between protein and gene
PTEN	0.4261	0.3401 to 0.5050	<0.0001	weak
EGFR	0.4673	0.3849 to 0.5424	<0.0001	weak
IGF1R	0.1583	0.05873 to 0.2548	0.0014	weak
mTOR	0.08514	-0.01555 to 0.1841	0.0878	weak
p-mTOR	0.02079	-0.07993 to 0.1211	0.6773	weak

TCGA Firehose Legacy: correlation between RNA seq and RPPA (N=844)

Biomarker	r	95% confidence interval	p value	correlation between protein and gene
PTEN	0.4752	0.4194 to 0.5274	<0.0001	weak
EGFR	0.5064	0.4527 to 0.5564	<0.0001	weak
IGF1R	0.1426	0.07374 to 0.2102	<0.0001	weak
MTOR	0.09405	0.02458 to 0.1626	0.0064	weak
pMTOR	0.1646	0.09608 to 0.2316	<0.0001	weak

CPTAC cell 2020: correlation between RNA seq and mass spectrometry-based proteomics (N=122)

Biomarker	r	95% confidence interval	p value	correlation between protein and gene
TOP2A	0.006011	-0.1771 to 0.1887	0.9476	weak
PTEN	0.5872	0.4530 to 0.6954	<0.0001	weak
EGFR	0.8305	0.7612 to 0.8810	<0.0001	strong
IGF1R	0.7409	0.6455 to 0.8136	<0.0001	strong
mTOR	0.3172	0.1418 to 0.4733	0.0004	weak

Table S8. Summary of signatures for predicting the patient outcome in breast cancer in the context of chemotherapy treatment and the reason they were not applicable to the Breast 300.
T/FAC (paclitaxel/fluorouracil/anthracycline/cyclophosphamide)

Study	Gene signature summary	Number of genes	Subtype	Main finding	Reason
Fu *et al.*[1]	Immune-associated 25-gene signature	25	All breast cancer	Using the LASSO method and partial likelihood deviance as the minimum criteria, the study identified an optimal 25-gene signature for predicting pCR to T/FAC neoadjuvant chemotherapy. This signature demonstrated a high predictive accuracy with an Area Under the ROC Curve of 0.9558.	23 of the 25 genes were available in the Breast 300 cohort. The normalisation step and weight step cannot be applied (data not available)
Liu *et al.*[2]	186-gene "invasiveness" gene signature (IGS)	186	All breast cancer	The IGS showed strong prognostic value in breast cancer, with significant associations with overall and metastasis-free survival ($p<0.001$, for both)	160 of the 186 genes were available in the Breast 300 cohort. The normalisation step and weight step cannot be applied(data not available)
Zhang *et al.*[3]	8 DNA Repair-Related Genes (DRGs)	8	All breast cancer	DRGs had a good predictive accuracy. The areas under the curve were 0.717 for 3-year survival and 0.772 for 5-year survival in the GSE9893 data set and 0.691 for 3-year survival and 0.718 for 5-year survival in the GSE42568 data set.	The weight step cannot be applied(data not available)
Sota *et al.* [4]	immune-related 23-gene signature for NAC (IRSN-23)	23	All breast cancer	Twenty-three out of 934 probes were selected for the signature based on their differential expression between pCR and non-pCR cases, along with cross-validation. The pCR rate among genomically predicted responders (Gp-R) was significantly higher than in non-responders (Gp-NR) (38% vs. 0%, $p = 1.04 \times 10^{-4}$). IRSN-23 emerged as the strongest predictor of pCR (odds ratio = 4.6, 95% CI= 2.7–7.7, $p = 8.25 \times 10^{-9}$) in the	Gp-R and Gp-NR were defined by hierarchical cluster analysis

				context of neoadjuvant chemotherapy.	
Turner *et al.* [5]	Consensus Signature (ConSig)	97	TNBC	The signatures were selected based on the mechanism of action of anthracyclines. Among various gene combinations, ConSig1 (STAT1 + TOP2A + LAPTM4B) demonstrated strong predictive ability for anthracycline resistance in a cohort of anthracycline-treated TNBC patients (NPV = 85%, PPV = 35%, OR = 3.18, p = 0.008).	93 of 95 genes were available in the Breast 300 cohort. The normalisation step and weight step cannot be applied(data not available)
Omar *et al.* [6]	Notch 5-TSPs signature	10	TNBC	Gene sets regulated by Notch signalling were retrieved from the Molecular Signature Database, and individual genes were extracted. The resulting model achieved an AUC of 0.80 and a precision-recall curve (PRC) value of 0.89 to predict patient outcome in the context of neoadjuvant chemotherapy.	k-TSPs model trained specifically was not available
Gamez-Pozo *et al.*[7]	PRM-predictor P5	4	TNBC	A reduced 5-protein signature was derived from an initial panel of 18 proteins with prognostic value. Among the combinations tested within this 5-protein panel, predictor P5 demonstrated the strongest performance in predicting relapse following adjuvant chemotherapy in TNBC patients. The 5-year distant metastasis-free survival (DMFS)with adjuvant chemotherapy was 63.54% in the low-risk group compared to 39.99% in the high-risk group (HR = 2.322, *p* = 0.0142).	The Breast 300 cohort does not include protein expression data for these biomarkers.

1. Fu, C., et al., *An Immune-Associated Genomic Signature Effectively Predicts Pathologic Complete Response to Neoadjuvant Paclitaxel and Anthracycline-Based Chemotherapy in Breast Cancer.* Front Immunol, 2021. **12**: p. 704655.
2. Liu, R., et al., *The prognostic role of a gene signature from tumorigenic breast-cancer cells.* N Engl J Med, 2007. **356**(3): p. 217-26.
3. Zhang, D., et al., *Prediction of Overall Survival Among Female Patients With Breast Cancer Using a Prognostic Signature Based on 8 DNA Repair-Related Genes.* JAMA Netw Open, 2020. **3**(10): p. e2014622.
4. Sota, Y., et al., *Construction of novel immune-related signature for prediction of pathological complete response to neoadjuvant chemotherapy in human breast cancer.* Ann Oncol, 2014. **25**(1): p. 100-6.
5. Turner, N., et al., *A multifactorial 'Consensus Signature' by in silico analysis to predict response to neoadjuvant anthracycline-based chemotherapy in triple-negative breast cancer.* NPJ Breast Cancer, 2015. **1**: p. 15003.
6. Omar, M., et al., *Notch-based gene signature for predicting the response to neoadjuvant chemotherapy in triple-negative breast cancer.* J Transl Med, 2023. **21**(1): p. 811.
7. Gamez-Pozo, A., et al., *Prediction of adjuvant chemotherapy response in triple negative breast cancer with discovery and targeted proteomics.* PLoS One, 2017. **12**(6): p. e0178296.
